# Comment on Montagnani et al. Optimization of RAASi Therapy with New Potassium Binders for Patients with Heart Failure and Hyperkalemia: Rapid Review and Meta-Analysis. *J. Clin. Med.* 2021, *10*, 5483

**DOI:** 10.3390/jcm11102755

**Published:** 2022-05-13

**Authors:** Donna Zarzuela, Alex Chin

**Affiliations:** Global Medical Affairs, AstraZeneca, Gaithersburg, MD 20878, USA; alex.chin@astrazeneca.com

The recent rapid review and meta-analysis by Montagnani et al. [1] evaluated the optimization of renin–angiotensin–aldosterone system inhibitor (RAASi) therapy with the new potassium binders (NPBs), sodium zirconium cyclosilicate (SZC) and patiromer, for patients with or at risk of heart failure (HF) and hyperkalemia. We wish to express our concern with the apparent prematurity of this analysis, and the limitations of the methodology and interpretation of the data, which appear to confer an overall favorable bias towards patiromer.

First, we question the timing of this meta-analysis; the optimization of RAASi therapy using NPBs is a highly active area of investigation, with recently completed but not yet published studies [2,3], and many others ongoing [4,5,6,7,8]. While the authors identified 25 ongoing study records, only 3 studies, lacking heterogeneity, with a limited time of follow-up (1–3 months), and with small numbers of events and patients, were included in the meta-analysis; we contend that this hinders the drawing of valid conclusions. One of the studies included was stopped early due to the coronavirus disease 2019 pandemic, resulting in a shift to exploratory endpoints (PRIORITIZE-HF) [2]. Importantly, data on long-term RAASi use from two international, Phase 3 clinical trials (HARMONIZE Open Label Extension [OLE] and ZS-005) demonstrating the safety and efficacy of SZC for up to 12 months, including in patients with HF [9,10], were also excluded from this analysis due to the lack of a comparator arm. Here, in 83 patients receiving RAASi at baseline in HARMONIZE OLE, RAASi use remained either stable or increased for most patients (90.3%) over the 11-month follow-up period, with only very few patients (3.6%) discontinuing RAASi [9]. Similarly, in 483 participants who received RAASi at baseline in ZS-005, 74% maintained the same dose over the 12-month follow-up period and 13% had a dose increase; a total of 14% of baseline RAASi-naive participants initiated RAASi therapy [10]. While these studies are not randomized clinical trials, they remain important registrational trials that contributed towards the regulatory approval of SZC, supporting the long-term utility of SZC for the management of hyperkalemia in individuals who would benefit from the continuation and optimization of RAASi. Excluding these studies has resulted in an imbalance in this overview of NPBs.

Second, the authors appear to apply unsubstantiated bias towards patiromer in their interpretation of the data. For example, the PRIORITIZE-HF trial (NCT03532009; a Phase 2 study evaluating SZC for RAASi optimization) [2] demonstrates a relative lack of risk of bias using the Cochrane Risk of Bias tool, consistent with other included studies. The authors employed raw data from the PRIORITIZE-HF trial which stopped early (N = 182 patients enrolled of 280 planned), so effect endpoints were underpowered and exploratory in nature. However, without providing rationale, the authors later state that PRIORITIZE-HF had a high risk of bias, resulting in exclusion from the sensitivity analysis.

Additionally, selective reporting of non-significant, directional concordances further strengthens the inappropriate bias towards patiromer. The authors also state: “Patiromer seems to have an effect on MRA optimization (high certainty of the evidence), while ZSC [SZC] seems to have no effect on optimizing MRA therapy (low certainty of evidence)”. Contrary to this, forest plots of patiromer and SZC for MRA optimization are directionally consistent with similar effect sizes, modestly in favor of both NPBs. Definitions of high, moderate, and low certainty of evidence appear to be based exclusively on 1) the slightly higher effect size of patiromer (risk ratio 1.25, 95% confidence interval 1.08–1.45) compared with SZC (1.19, 0.89–1.59)—a difference in effect size of 0.06—and 2) the lower bounds of the two patiromer studies fractionally exceeding 1. Whilst the similarity of the effect size of SZC and patiromer on MRA optimization is ignored, a statistically insignificant but directionally consistent effect of patiromer on angiotensin-converting enzyme inhibitor/angiotensin receptor blocker therapy (ACEi/ARB) optimization is noted in the discussion, described as being in accordance with the (significant) MRA outcome with patiromer. However, little regard appears attributed to the quality of this evidence, which is simply reported as “very low certainty for imprecision and risk of bias”.

The inappropriate data interpretation is also evident later regarding an apparent biased and uncontextualized interpretation of safety data for the NPBs. For example, adverse events (AEs) for patiromer in patients with HF are reported as being similar to those in patients with hyperkalemia; yet, when highlighting chronic cardiac failure as an AE associated with SZC from the PRIORITIZE-HF trial, they do not note that the number of HF-worsening serious AEs with SZC was similar to placebo. In fact, the safety profile of SZC in HF patients in PRIORITIZE-HF was consistent with that from other SZC clinical trials, with no signal of increased risk of HF worsening or edema [2]; full safety data and context surrounding these results will be included in the imminent PRIORITIZE-HF publication. In addition, although both NPBs are associated with hypokalemia (in clinical trials of patiromer and SZC, ~4.7% and 4.1% of patients, respectively, developed hypokalemia) [11,12], the authors only note the association of SZC with hypokalemia and do not mention the same for patiromer.

The following statement is also misleading: “The effect, quality of evidence, and less damaging AEs in long-term use seem to suggest the use of patiromer for the optimization of MRA therapy in patients with or at risk of HF and hyperkalemia”. The effect of SZC on MRA optimization is directionally consistent with patiromer, suggesting that neither NPB holds a significant advantage in contrast to this statement. Regarding the quality of evidence, subjective levels of certainty are given significant weight throughout this analysis. Reference to there being “… less damaging AEs in long-term use …” with patiromer is also unsubstantiated; the long-term safety of SZC was demonstrated for up to 1 year in HARMONIZE OLE and ZS-005 as alluded to earlier [9,10]. In addition, although the authors acknowledge that the effects of potassium binder interventions on longer-term outcomes were uncertain, this important limiting caveat does not appear to have fed into their conclusion. The authors conclude their report by stating their conclusions are supported by other systematic reviews. They refer to Shrestha and colleagues [13], with no mention of the associated letter to the editor published in response [14], which expressed concern over the risk of inaccurate and misleading conclusions due to the omission of important datasets for SZC.

In summary, the limitations of the methodology and data interpretation inappropriately confer an overall favorable bias towards patiromer. International Phase 3 clinical trials have demonstrated the safety and efficacy of SZC for up to 12 months among patients with multiple comorbidities, including chronic kidney disease, HF, and diabetes mellitus, the majority of whom were also receiving RAASi therapy [9,10]. The safety profile of SZC in patients with HF in PRIORITIZE-HF was also consistent with that from other SZC clinical trials. Finally, the 2021 European Society of Cardiology Guidelines, the 2021 American College of Cardiology Expert Consensus Decision Pathway, and the 2020 and 2021 Kidney Disease: Improving Global Outcomes Clinical Practice Guidelines, include both SZC and patiromer as treatment options for the management of hyperkalemia in patients with HF and/or chronic kidney disease, to allow the initiation and uptitration of guideline-directed RAASi therapy in more patients [15,16,17,18].
